# Safely Managed On-Site Sanitation: A National Assessment of Sanitation Services and Potential Fecal Exposure in Indonesia

**DOI:** 10.3390/ijerph18158204

**Published:** 2021-08-03

**Authors:** Mitsunori Odagiri, Ann Thomas, Maraita Listyasari, Freya Mills, Robert E. S. Bain, Zainal Muhammad, Tom Slaymaker, Aldy Mardikanto, Anita Gultom, Asri Indiyani, Hasnani Rangkuti, Juliet Willetts

**Affiliations:** 1 United Nations Children’s Fund (UNICEF), Jakarta 12920, Indonesia; anthomas@unicef.org (A.T.); mlistyasari@unicef.org (M.L.); mzainal@unicef.org (Z.M.); 2 Institute for Sustainable Futures, University of Technology Sydney, Ultimo, NSW 2007, Australia; freya.mills@uts.edu.au (F.M.); Juliet.Willetts@uts.edu.au (J.W.); 3 Division of Data, Analysis, Planning and Monitoring, United Nations Children’s Fund (UNICEF), New York, NY 10017, USA; rbain@unicef.org (R.E.S.B.); tslaymaker@unicef.org (T.S.); 4 National Development Planning Agency (Bappenas), Government of Indonesia, Jakarta 12920, Indonesia; aldy.mardikanto@bappenas.go.id; 5 Ministry of Health, Government of Indonesia, Jakarta 12950, Indonesia; anitarentauli@yahoo.com; 6 Ministry of Public Work and Housing, Government of Indonesia, Jakarta 12110, Indonesia; asri_indiyani@yahoo.com; 7 National Bureau of Statistics (BPS), Government of Indonesia, Jakarta 10710, Indonesia; hasnani@bps.go.id

**Keywords:** on-site sanitation, safely managed services, inequalities, fecal exposure, Indonesia, sustainable development goals

## Abstract

Sustainable Development Goal target 6.2 calls for universal access to adequate and equitable sanitation, setting a more ambitious standard for ‘safely managed sanitation services’. On-site sanitation systems (e.g., septic tanks) are widely used in low- and middle-income countries (LMICs). However, the lack of indicators for assessing fecal exposure risks presents a barrier to monitoring safely managed services. Furthermore, geographic diversity and frequency of disasters require a more nuanced approach to risk-informed decision-making. Taking Indonesia as an example, the purpose of this paper is to provide insights into current status and practices for on-site sanitation services in the contexts of LMICs. Using a dataset from a national socio-economic survey (*n* = 295,155) coupled with village census (*n* = 83,931), we assessed (1) household sanitation practices across Indonesia stratified by city-level population density and meteorological factors, (2) factors associated with septic tank emptying practice, and (3) inequalities in potential fecal exposure as measured by population density and WASH access by wealth quintile. We found a high reliance on on-site sanitation facilities (80.0%), almost half of which are assumed to be ‘uncontained’ septic tanks and one in ten facilities discharging untreated waste directly into the environment. The most densely populated areas had the highest rates of septic tank emptying, though emptying rates were just 17.0%, while in the lowest population density group, emptying was rarely reported. Multivariate regression analysis demonstrated an association between flooding and drought occurrence and septic tank emptying practice. Higher groundwater usage for drinking among poorer households suggests unsafe sanitation may disproportionally affect the poor. Our study underscores the urgent need to strengthen the monitoring of on-site sanitation in LMICs by developing contextualized standards. Furthermore, the inequalities in potential fecal exposure require greater attention and tailored support mechanisms to ensure the poorest gain access to safely managed sanitation services.

## 1. Introduction

The Sustainable Development Goal (SDG) targets 6.1 and 6.2 call for the elimination of open defecation and universal access to drinking water, sanitation, and hygiene (WASH), addressing the unfinished business of the Millennium Development Goals (MDGs) and setting ambitious new service norms for drinking water and sanitation [1]. The criteria for a ‘safely managed’ sanitation service (SDG 6.2) go beyond access to improved sanitation (which is designed to hygienically separate excreta from human contact) with a focus on safe excreta management across the entire sanitation service chain [1]. On-site systems that are not properly sited, designed, installed, and maintained pose an unacceptable risk to public health. According to the Joint Monitoring Programme (JMP), a safely managed sanitation service means that people should use improved sanitation facilities that are not shared with other households, and the excreta produced should be treated and disposed in situ, stored temporarily, and then emptied and transported to treatment off-site or transported through a sewer with wastewater and then treated off-site. The ambition for a higher level of service-through technical improvement of existing sanitation systems or in the design and the implementation of new ones to reduce these public health risks is supported by the conclusions of recent WASH trials [2] and the development of tools such as the Shit/Excreta Flow Diagram [3] and the Pathogen Hazard Diagram [4].

Globally, an estimated 3.4 billion people or 45% of the population used safely managed sanitation services in 2017 [5]. However, data availability is limited, and national estimates were only available for 96 countries, representing 54% of global population [5]. Furthermore, while many countries had national data on treatment of wastewater from households connected to sewers, very few had data on treatment and disposal of waste from on-site facilities. This remains the single biggest data gap for global monitoring of the SDG sanitation target [1,6,7]. Other areas where there is missing or poor quality data include sanitation system typologies, emptying practices in both urban and rural areas, and the ability to assess the magnitude of the fecal exposure risks associated with the appropriate management of such on-site systems. There are further data gaps around the influence of weather conditions on on-site sanitation management, which are altered further by climate change [8,9,10,11];, knowledge of these impacts is limited to small scale study areas [12,13]. 

Taking Indonesia as an example, the purpose of this paper is to address these gaps by providing insights into the current status and practices for on-site sanitation services in the contexts of LMICs. Indonesia made extensive progress in the reduction of open defecation practices [14] at an annual rate of 1.3 percentage points per year [5]. Yet, approximately 20 million people still practice open defecation [5,15]. As sewer connections remain limited nationally (maximum 2%) and coverage changed little in recent years [16], most households rely on on-site sanitation systems [5]. In Indonesia, septic tanks, leach pit latrines, and lined pit latrines are widely used by households [4,17,18]. The term ‘septic tank’ is widely used in Indonesia to refer to all on-site systems including those that are not technically septic tanks [3,17]. In a watertight septic tank, the liquid effluent can only leave through the effluent pipe; there is no leakage. Thus, a watertight septic tank requires regular desludging to maintain functionality. A ‘leaking’ septic tank or a leach pit latrine requires less frequent sludge removal but may pose a health risk in areas where shallow groundwater is used for drinking [19]. This underscores the need for a better understanding of on-site sanitation systems and fecal sludge management practice across the country to inform national water and sanitation strategies. 

We analyzed a unique dataset of a large nationally representative survey (the Indonesian national socio-economic survey, known as Susenas) that was collected in 2018 (*n* of households = 295,155) coupled with an Indonesian village census data on extreme climate event occurrence (*n* of villages = 83,931) to respond to the following key objectives: (i) to examine the current patterns of household use of a sanitation facility and emptying of on-site sanitation facilities across Indonesia based on population density and meteorological factors; (ii) to explore factors associated with the household septic tank emptying practices; and (iii) to assess inequalities in potential fecal exposure as measured by population density and WASH access by wealth quintile. Notably, we attempted to estimate coverage of ‘contained’ and ‘uncontained’ septic tanks separately based on the best available data. Finally, we examined the wider implications on safely managed sanitation programming and monitoring in Indonesia and LMICs with similar contexts in support of achieving SDGs targets.

## 2. Materials and Methods

### 2.1. Data Source and Overall Analysis

Data from a national socio-economic household survey (known as Susenas) collected by the National Bureau of Statistics, Indonesia in March 2018 (*n* of households = 295,155) were used for the analysis. Further details of the survey design can be found elsewhere [20]. The survey collected self-reported information on whether the household either has: no sanitation facility (i.e., open defecation), the type of sanitation facility used by family members, or use a public/communal facility. Information was also collected on the disposal of fecal waste from household sanitation systems, including sewer, septic tank (contained and uncontained), pit (unlined), or direct discharge into the open environment without any treatment (e.g., a pour-flush latrine and pit latrine connected to river, lake, rice field, and beach). Households were also asked whether or not their septic tank was emptied in the past five years; the maximum period of use before a septic tank built to Indonesian standards requires emptying [21]. 

In Indonesia, most households call their on-site sanitation systems a ‘septic tank’, including those that are not watertight [18,22]. For the purpose of this study, it was necessary to deduce whether the household had an effectively functioning septic tank (that meets design and construction criteria) or an uncontained tank (i.e., one that is not watertight). We deduced this based on the reported age of the septic tank and whether it was emptied in the last 5 years. Thus, septic tanks were classified into four sub-groups: (1) septic tanks that were emptied at least once; (2) septic tanks where it was unknown whether they were emptied or not; (3) septic tanks less than 5 years old, which were not emptied; and (4) septic tanks 5 or more years old, or age unknown, that were not emptied. 

In this study, we considered that sub-groups (1) and (3) met the technical specifications for effectively functioning septic tanks, and we refer to them as ‘contained’ septic tanks. We term sub-group (4) as ‘uncontained’ septic tanks, since they are not fully watertight. Sub-group (2) was excluded from this ‘contained’ and ‘uncontained’ septic tank classification. Our septic tank classification system is further described in the flowchart in the Supplemental Materials (Figure S1).

Our unit of analysis was municipalities (*n* = 99) or districts (*n* = 415), and household weighted descriptive statistics on sanitation facility types were produced for each municipality and district. Municipalities and districts were grouped by population density rather than a binomial urban vs. rural classification (see Table S1). This is because the interaction between population density and sanitation has significant health impacts and thus provides a clearer picture of the challenges, including technologies choices [23]. Population density for each district and municipality was estimated based on the population projection data (known as SUPAS Projection Data, BPS). All 514 municipalities/districts were then grouped into four categories: over 10,000 people/km^2^ (*n* = 14), 1000–10,000 people/km^2^ (*n* = 94), 100–1000 people/km^2^ (*n* = 189), and fewer than 100 people/km^2^ (*n* = 217). Additionally, given that meteorological factors could potentially affect septic tank filling rates and emptying practices [8,13], proxy indicators (i.e., occurrence of flooding and drought over the last 3 years) were selected in this analysis [24]. As a proxy for likelihood of extreme climate event occurrence, village census data (known as PODES, 2018) collecting experience of flooding and drought over the last 3 years in each village (*n* of villages = 83,931) were aggregated at municipality and district level, resulting in the proportion of villages (known as desa for districts or Kelurahan for municipalities in Indonesia) experiencing flooding and the proportion experiencing drought. Similar to population density categories, 514 municipalities/districts were grouped into four categories; over 75% of villages experienced flooding (*n* = 6) or drought (*n* = 0), 50%–75% of villages experienced flooding (*n* = 59) or drought (*n* = 4), 25%–50% of villages experienced flooding (*n* = 156) or drought (*n* = 48), and less than 25% of villages experienced flooding (*n* = 293) or drought (*n* = 462).

### 2.2. Assessment of Inequalities in Potential Fecal Exposure

We examined two types of inequalities associated with potential fecal exposure. First, among the survey indicators collected, open defecation, direct discharge into environment from sanitation facilities, and ‘uncontained’ septic tanks are evident public health risks exacerbated by population density [25]. While open defecation and direct discharge from sanitation facilities could pose a health risk due to fecal exposure as well as through contaminated drains, surfaces, food chain, and potential ingress into water supply from the surface (i.e., surface pathways), ‘uncontained’ septic tanks could adversely impact groundwater quality depending on the context (i.e., sub-surface pathways). Estimates were made based on each municipality/district population density and the proportion of households reporting each form of inadequate sanitation (open defecation, direct discharge, ‘uncontained’ septic tanks) as a proxy of quantitative fecal loads released into the environment. We assumed that variation of household size within each municipality and district was negligible (i.e., municipality and district level average household size was used). Open defecation, sanitation facility discharging directly into the environment, and ‘uncontained’ septic tank densities were then compared across municipality/district population density categories.

Secondly, we examined wealth-associated inequalities in potential fecal exposure as measured by selected water and sanitation variables across population density categories. We chose ‘practicing open defecation’, ‘use of a sanitation facility directly discharging human waste into open environment’, ‘use of a septic tank that was not emptied over the last 5 years’, ‘use of groundwater for drinking source’, and ‘groundwater with presence of a septic tank within 10 m from the water source’ as potential fecal exposure pathways [26,27]. Impacts of the distance between a septic tank and groundwater source on fecal contamination varies and depends on context specific parameters such as soil types and groundwater table depth [19]. In this analysis, we compared households with and without a septic tank within 10 meters from the (groundwater) source. These indicators were compared between the poorest (bottom 20%), the mean, and the richest (top 20%) households within each population density category. The wealth quintiles were created based on expenditure per capita following the national statistical office’s approach.

### 2.3. Statistical Analysis

A conceptual model of multi-level factors influencing household septic tank emptying practice was developed to guide the selection of factors a priori (see Supplemental Material Figure S2.). Three levels of factors were considered: factors associated with (1) municipality and district-level environments, (2) household socio-economic status, and (3) sludge accumulation at facility level. The primary outcome was self-reported household septic tank emptying practice (i.e., at least once over the last five years). Households reporting use of a septic tank were included in this analysis (*n* = 136,225). We used Poisson regression (log-link) with generalized estimated equations (GEE) and robust standard errors, adjusting for municipality and district-level clustering, to estimate adjusted prevalence ratios (aPR) [28].

To assess correlation between municipality/district characteristics (i.e., population density and meteorological factors) and reported septic tank emptying practices as well as open defecation prevalence, Spearman rank correlation coefficients and associated test statistics were calculated.

All of the analyses were conducted in SPSS ver. 22 (SPSS Inc., Chicago, IL, USA). In all of the analyses, *p* < 0.05 was considered significant.

## 3. Results

### 3.1. Household Sanitation Facility Use and Emptying of On-Site Sanitation Facilities

**Figure 1:**
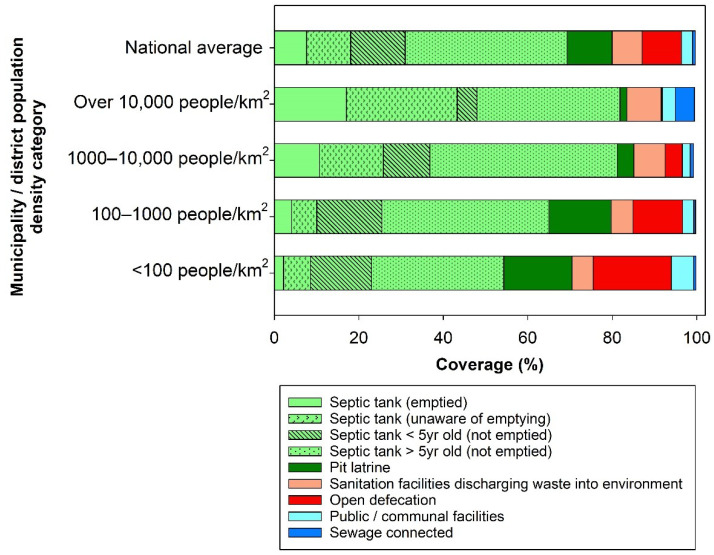
Household sanitation facilities use and emptying across four population density categories at municipality and district level (over 10,000 people/km^2^: *n* = 14, 1000–10,000 people/km^2^: *n* = 94, 100–1000 people/km^2^: *n* = 189, <100 people/km^2^: *n* = 217). Note that, given local contexts and best available data, septic tank age and emptying practice over the last 5 years were used to differentiate between ‘contained’ and ‘uncontained’ septic tanks. The item ‘septic tanks equal to or over 5 years old (not emptied)’ includes the item ‘septic tank age unknown (not emptied)’ and was used as a proxy for ‘uncontained’ septic tanks.

We observed notable patterns of sanitation facility usage and septic tank emptying practice in different population density categories of municipalities and districts in Indonesia (Figure 1). All municipalities and districts showed high rates of reliance on on-site sanitation systems (80% nationally) including ‘septic tanks’ (both ‘contained’ and ‘uncontained’ tanks) and ‘pit latrines’. In the most densely populated municipality/district category, the highest sewerage access (4.5%), the highest septic tank use (81.8%), and the lowest open defecation rate (0.2%) were reported. Conversely, in the lowest population density category, the highest open defecation rate (18.5%), the highest pit latrine usage (16.2%), and the lowest septic tank use (54.3%) were reported. The proportion of households using a sanitation facility directly discharging human waste into the environment ranged from 5.0% to 8.2% of households in each population density category.

With regards to emptying practice, the highest proportion of both ‘emptying’ and ‘unaware of emptying’ responses was reported in the most densely populated category (17.0% and 26.2%, respectively). In contrast, emptying practice was rarely reported in the lowest population density category (2.6%). Based on a combination of septic tank age and emptying practice data, more than half of self-reported septic tanks were estimated to be ‘uncontained’ nationally. The highest ‘uncontained’ septic tank use was reported in the second most dense group (44.4%).

**Figure 2:**
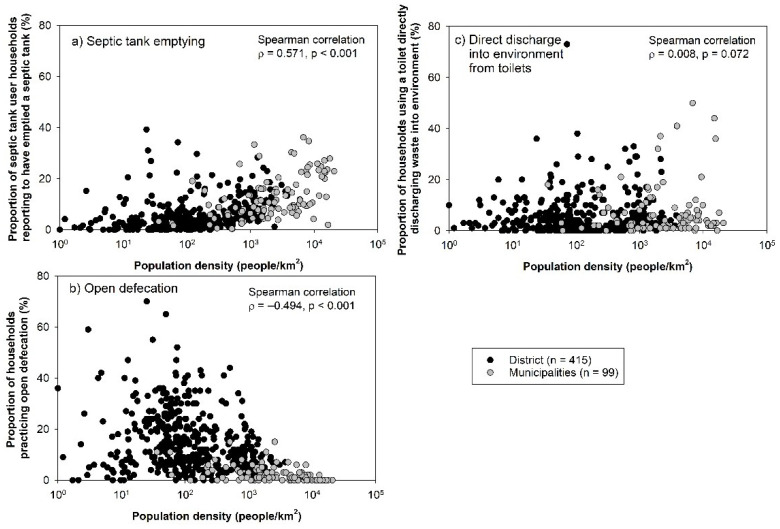
Proportion of households using septic tanks that were emptied (**a**) (**top left**), practicing open defecation (**b**) (**bottom left**), and using sanitation facilities that directly discharge human waste to environment (**c**) (**top right**) by population density. Each dot represents one municipality (grey circle, *n* = 99) or district (black circle, *n* = 415).

We found a significant positive correlation between the proportion of households having emptied a septic tank and population density (Figure 2a, σ = 0.57), whereas there was an inverse relationship between open defecation prevalence and population density (Figure 2b, σ = −0.49). No correlation was found between levels of direct discharge to the environment from sanitation facilities and density categories (Figure 2c).

### 3.2. Factors Associated with Household Reported Emptying Practice

**Figure 3:**
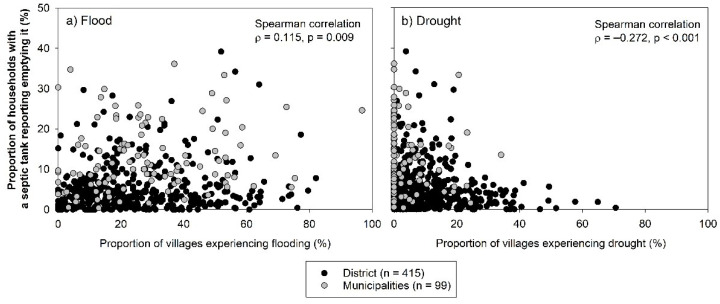
Proportion of households reporting septic tank emptying over proportion of villages experiencing flooding (**a**) (**left**) and proportion of villages experiencing drought (**b**) (**right**). Each dot represents one municipality (grey circle, *n* = 99) or district (black circle, *n* = 415).

In multivariate analysis, almost all tested factors were found to be significantly associated with self-reported septic tank emptying practices except for administrative unit (Table 1). The largest adjusted prevalence ratio (aPR) was related to septic tank age (more than 10 years relative to less than 5 years, aPR 5.20, 95% CI 4.67–5.80 followed by population density categories and wealth quintile. Whereas greater flood occurrence was associated with increased prevalence of emptying practices, the reverse was true for drought occurrence. In comparison to regions where <25% of villages were affected, regions where >75% of villages experienced flooding were associated with 40% increase of emptying practice prevalence (aPR 1.40, 95% CI 1.06–1.85), and regions where >75% of villages experienced drought were associated with an 86% decrease (aPR 0.14, 95% CI 0.02–1.23). Consistent with findings of the multivariate analysis, a significant but weak correlation was observed between proportion of households having emptied a septic tank and flood and drought occurrences (Figure 3a flooding; ρ = 0.12) and Figure 3b drought; ρ = −0.27).

### 3.3. Inequalities in Potential Fecal Exposure

**Figure 4:**
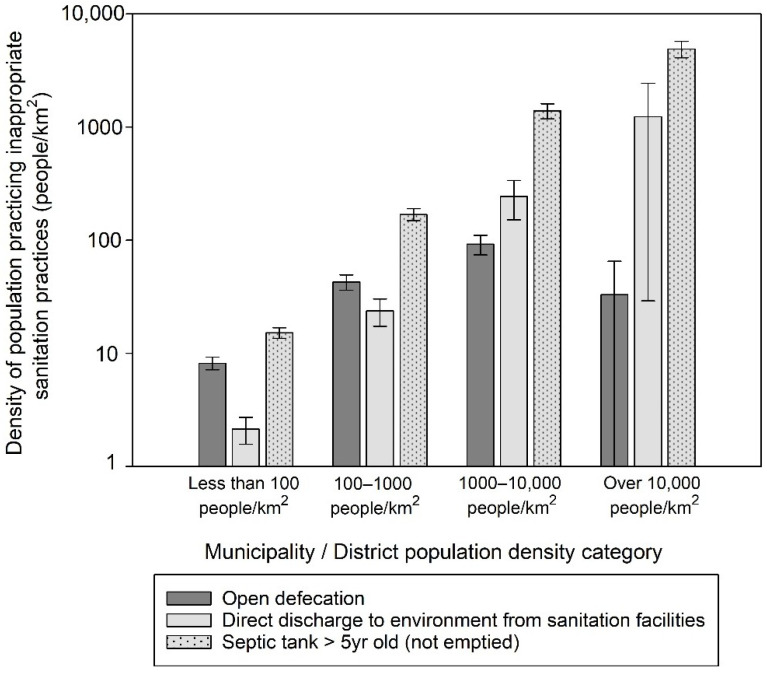
Average population density of open defecation, direct discharge of human waste into environment from sanitation facilities, and septic tanks over 5 years old that were not emptied (a proxy for ‘uncontained’ septic tanks), respectively, over population density categories of municipalities and districts (over 10,000 people/km^2^: *n* = 14, 1000–10,000 people/km^2^: *n* = 94, 100–1000 people/km^2^: *n* = 189, <100 people/km^2^: *n* = 217). Error bar represents 95% confidence interval of the mean.

To assess potential fecal exposure, the average densities of open defecation, direct discharge of human waste from sanitation facilities, and ‘uncontained’ septic tanks were estimated across four population density categories of municipalities and districts (Figure 4). Looking at surface contamination pathways, the second most dense areas showed high density levels of both open defecation and discharge of human waste from sanitation facilities directly into the environment, while in the most densely populated areas, direct discharge from sanitation facilities was common. In sub-surface contamination pathways, we found remarkably high ‘uncontained’ septic tank density (over 1000 people/km^2^) in the first and the second most dense areas.

**Figure 5:**
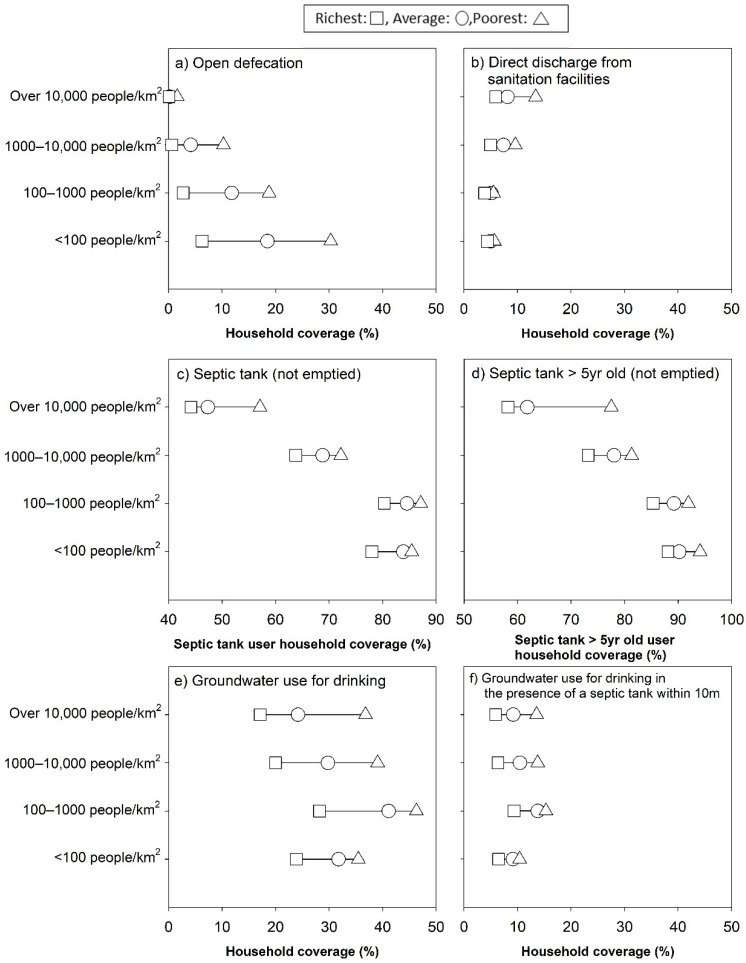
Inequalities in the proportion of households with potential fecal exposure pathways including (**a**) open defecation, (**b**) use of a sanitation facility that discharges human waste directly into the environment without treatment, (**c**) use of a septic tank that was not emptied, (**d**) use of a septic tank that is over 5 years old and was not emptied (‘uncontained’ septic tank), (**e**) use of groundwater for drinking, and (**f**) use of groundwater for drinking with a septic tank within 10 m from the water source. Note that, for (**c**), the denominator was the number of households using a septic tank, while for (**d**), it was the number of households using a septic tank that is over 5 years old. Square, circle, and triangle denote “richest”, “average”, and “poorest” quintiles, respectively. The scale of all six figures (**a**–**f**) is the same despite different ranges.

The assessment of wealth-associated water and sanitation access found substantial inequalities between the richest and the poorest households (Figure 5). Notably, the gap of open defecation practice between the richest and the poorest quintile increased as population density categories became less dense. Conversely, in the most densely populated category, larger percentage point differences between the richest and the poorest were observed in direct discharge and poor septic tank emptying practice. For groundwater use as a primary source of drinking water, considerable inequalities were found between wealth quintiles across all population categories of municipalities and districts. Among households using groundwater for drinking, the differences in presence of a septic tank within 10 m from the water source were less marked.

## 4. Discussion

Improving access to safely managed sanitation services and eliminating open defecation are central to SDG target 6.2 and many of LMICs’ national priorities. This study assessed current patterns of sanitation use and emptying practices, taking Indonesia as an example to inform LMICs’ on-site sanitation strategies.

Despite the high proportion of self-reported septic tank use in the national survey, the data show that, across urban and rural Indonesia, the majority of this most likely refers to ‘uncontained’ septic tanks. This underscores the need for further assessment of their safety including containment, emptying, and disposal of waste from on-site sanitation systems. Household surveys can provide valuable information on the types of facilities used and the frequency of emptying, which can be used to identify inequalities in risks associated with key fecal exposure pathways. However, additional information is needed to assess other exposure pathways. Technical inspections would be useful to determine the proportion of ‘contained septic tanks’ and ‘uncontained septic tanks’ that are appropriately designed and constructed to contain and treat wastes in-situ and the proportion that are broken, full, or leaking waste into the household environs. Service providers can provide information on transport, treatment, and disposal of waste that are removed from the household premises. This reaffirms the importance of systematic monitoring that goes beyond household data collection and methodological development with a combination of the household survey, administrative data, and technical inspections [6].

High reliance on ‘uncontained’ septic tanks is worrying in the context of Indonesia, as groundwater is the predominant source of drinking water for over 90% households in both urban and rural areas [29]. ‘Uncontained’ septic tanks could pose a risk to public health if the degree of separation from groundwater and pathogen attenuation in the sub-surface are insufficient [4,19,30,31]. This research also found that groundwater was used for drinking water across all population densities, particularly by the poor (Figure 5e). Child diarrhea was previously associated with consumption of microbiologically unsafe groundwater [32,33,34], suggesting poorer households may have higher diarrheal disease burden due to their greater exposure to contaminated groundwater. Surprisingly, almost irrespective of population densities, there are approximately 10% of households using groundwater for drinking water that have a septic tank within 10 m from their well (Figure 5f). This points to the need for strengthening regular, local monitoring systems such as those for water quality testing [19] while strengthening local government’s enforcement of the Indonesian National Sanitation Code and the Building Code related to septic tank design and construction. While mixed evidence exists on the relative contribution of on-site sanitation facilities to fecal contamination pathways of groundwater [35,36], high population density as a risk factor should not be discounted [35,37].

Widespread use of ‘uncontained’ septic tanks in Indonesia is most likely due to people’s preference, perceptions on what constitutes a ‘good’ septic tank, and a lack of awareness of the adverse impacts of poor on-site sanitation systems on public health and the environment. A recent sanitation market assessment in six urban cities of Indonesia reported that households commonly perceived a good septic tank to be one that never has to be emptied, with these ‘uncontained’ systems also found to be lower cost compared to effectively functioning septic tanks. Additionally, households using an ‘uncontained’ septic tank showed limited awareness about the potential adverse impact of poor sanitation [38]. Our finding that the poorest were unlikely to install and empty a septic tank also indicates that there is an economic barrier to doing so. Lack of awareness about potential health and environmental implications from unsafe sanitation facilities and sludge disposal was reported elsewhere [39,40]. While one sanitation market assessment [38] found that suppliers knew about the potential public health risks associated with ‘uncontained’ tanks, they reported that their business is driven by customer preference, indicating the need for effective communication to improve the perception on safe sanitation and raise awareness among the public. While standards for desludging frequencies vary across countries, ranging from 2 to 5 years [41], regular desludging of septic tanks is necessary to ensure functionality of the systems and prevent environmental pollution [8]. Moreover, a recent study estimated that half of on-site sanitation facilities are manually emptied in LMICs [42], pointing to the need for safe emptying practices to achieve safely managed sanitation. Finally, to address these barriers at household and service-provider levels, local government must enhance regulation and inspection for the quality of on-site sanitation systems [22].

This study showed that potential fecal exposure from direct discharge into the environment without treatment is unacceptably high in high-density urban areas, particularly for the poor (Figure 4 and Figure 5b). Direct discharge from sanitation facilities into drains and the environment remains an issue in Indonesia and elsewhere [3,43,44]. Flooding of open drains in urban environments could increase risks of pediatric enteric infection [44]. Additionally, a higher density of fecal loading as measured by the density of septic tank systems, contained/uncontained onsite sanitation facility, and the amount of human fecal loading into pour-flush latrine pits were found to be associated with increased diarrhea incidence in the U.S. [32], *Escherichia coli* concentration in drains in urban Ghana [45], and pathogen detection in tubewells in India, respectively [46]. Given that both high levels of flooding risk and high fecal exposure exist in urban centers of Indonesia, corrective actions coupled with systematic monitoring efforts are needed.

While sewerage systems in Indonesia are of limited scale (0.7% nationally), there exist more than 25,000 communal scale sanitation systems, which could offer an alternative in dense living environments. However, these tend to be limited to anaerobic baffled reactors, which only achieve partial pathogen removal [43], hence further treatment technologies are needed before effluent is safely discharged. Sustainability of these systems also requires appropriate governance systems, including adequate community mobilization and cohesion to manage and oversee operations—something often missing in culturally diverse low-income urban settlements. The technical and the financial requirements for maintaining and repairing these systems are often beyond the capacity of low-income communities and require local government support [47]. In this respect, sanitation sustainability indices could be a useful tool to evaluate and select appropriate sanitation systems from public health, economic benefit, and social acceptance standpoints for greater sustainability in communities [48]. Sustainability indices could be used to visualize trade-off between public health risks and economic gains, helping policy makers raise awareness about unsafe sanitation systems among public.

In rural low-density areas, high open defecation prevalence and low emptying practice suggest, while elimination of open defecation is still the top priority, a reconsideration of safely managed sanitation service standards is needed to inform sanitation investment choices. This includes on-site treatment or less frequent emptying options to reduce the vulnerability of the poorest, since provision of regular desludging services might be challenging and economically unviable in these settings [49]. It is reported that, in such areas, due to a lack of affordable emptying services, rural households tend to choose unhygienic manual emptying and dumping without adequate treatment such as flooding out (i.e., intentional release of sludge into the neighborhood during rainy seasons) or even reversion to open defecation [13,40,50,51]. This suggests that, despite lower potential fecal exposure as measured by the density metrics, public health risks are unlikely to be negligible, and further research is needed to better understand current fecal sludge management (FSM) practices and to assess the suitability of options for low-cost FSM in low population density areas.

Moreover, additional analysis indicates that it cannot be assumed that reduction of open defecation practices would always lead to increased emptying practices (Figure S3). Leveraging on-going efforts to eliminate open defecation, inclusion of FSM into national community-led total sanitation (CLTS) monitoring (i.e., post-triggering or post-open defecation free monitoring) could be useful to address this issue from the onset of sanitation program implementation [51] while supporting the poorest with FSM through targeted subsidies.

The observed effects of flooding and drought on septic tank emptying practice underscores that climate impacts should be incorporated as part of sanitation planning and decision-making processes in disaster-prone countries such as Indonesia. This is particularly true given the large number of villages experiencing extreme weather events observed in this study (flood: *n* of villages = 19,675 (23.4%), drought: *n* of villages = 8587 (10.2%)). Yet, oftentimes, the sanitation sector sees climate change as a low priority and does not yet proactively incorporate such risks into strategies and planning [11,24]. The positive association between septic tank emptying practice and flooding occurrence is consistent with previous studies in Cambodia, Tanzania, and elsewhere [8,12,52]. Heavy rainfall can cause overflowing of on-site sanitation facilities [8], while, during the rainy season, unhygienic pit latrine emptying or ‘flooding out’ to empty pits was reported elsewhere [50,52].

Both unintentional and intentional fecal sludge release could pose serious public health risks in urban and rural areas, hence climate-sensitive FSM planning and more options for desludging services and on-site systems/technologies are needed in disaster-prone areas. Intense flooding could also raise the groundwater table, exacerbating drinking water source contamination from ‘uncontained’ on-site sanitation [24], which would be of primary concern in densely populated areas of Indonesia, where the poor rely on groundwater for drinking. This suggests that the adverse impact would disproportionally affect poor and vulnerable groups, highlighting the need for action from an equity standpoint [11]. Conversely, drought occurrence was negatively associated with septic tank emptying, suggesting smaller sludge accumulation rates potentially due to limited water usage for flushing by households (i.e., lower water content in sludge) and higher temperature (i.e., acceleration in the microbiological digestion process) in these areas. It is also plausible that limited water access during drought could lead to reversion to open defecation, as observed in rural Indonesia [53].

There are substantive inequalities in potential fecal exposures from on-site sanitation facilities as measured by population density and water and sanitation access in Indonesia, suggesting the importance of viewing sanitation as both a private and a public good. While various intra-community and external support instruments should be in place to assist in reaching disadvantage groups [54,55], more importantly, national and local government roles, responsibilities, and accountability mechanisms should be enhanced to address such inequalities. An emerging framework and strategies such as the citywide inclusive sanitation public service approach [56] and inclusive programming [57] in urban and rural contexts, respectively, outline ways to address these roles and accountabilities in detail.

Finally, while measuring household septic tank emptying practice via a national household survey is an important first step to track on-site safely managed sanitation service chains, there is room to improve data collection. We observed a high response rate of ‘unknown’ for the question on emptying practice, particularly in the highest density areas. A similar challenge was reported in a previous study in Belize [58]. Additionally, there are challenges with terminology, as people use the term ‘septic tanks’ to refer to a wide range of sanitation system types in Indonesia [22]. This may indicate the need for better question formulation and/or better targeting of respondents responsible for sanitation facility management in the survey. However, in urban, highly populated areas, households may still be unlikely to know the type of system that is buried underground, especially if they share an on-site sanitation facility with other families and/or move backward and forward between urban and rural areas. We found the proportion of households that were unaware of emptying practice to be significantly higher among users of shared rather than private sanitation facilities (27% vs. 14%, χ^2^ test, *p* < 0.05), which illustrates the complexity of data collection.

The study had several limitations. First, the national socioeconomic survey provided no data on the fate of fecal sludge after it is removed off-site and therefore is unable to shed light on the safety of the entire on-site sanitation service chain. Secondly, in Indonesia, the terms ‘septic tank’ and ‘uncontained septic tank’ (i.e., septic tanks that are not watertight) are used interchangeably, resulting in difficulty in differentiating between on-site sanitation types [22]. In an effort to clarify this point, we differentiated between ‘contained’ and ‘uncontained’ septic tanks in this study using a combination of the available data (i.e., septic tank age and emptying practice accounting for the advised emptying frequency of 5 years). This classification is imperfect not least because it assumes that all recently installed and recently emptied systems (<5 years) are ‘contained’ septic tanks rather than ‘uncontained’ septic tanks. Other factors such as the size of the tank are known to affect the emptying frequency but were not captured in the household survey.

## 5. Conclusions

In this study, we assessed sanitation usage, emptying practice, and inequalities in fecal exposures across 514 municipalities and districts in Indonesia. We found widespread reliance on on-site sanitation, and this study suggests almost half of these systems are ‘uncontained’ septic tanks, i.e., leaking septic tanks. Many sanitation facilities discharge directly into the environment without treatment, pointing to the need for enhanced monitoring of the safety of on-site sanitation and drinking water quality. Given that emptying of on-site systems was rare in the lowest population density areas, contextualized standards for safely managed services or less frequent emptying options for rural areas are needed to fully reduce geographic inequalities. Extreme weather events such as floods and droughts are also associated with emptying practices, implying that climate risks should be considered in sanitation planning and underscoring the need for a more nuanced understanding of people’s coping mechanisms (i.e., FSM behavior) during extreme weather events.

In higher population density areas, the greatest potential fecal exposure is related to a combination of direct discharge, ‘uncontained’ septic tanks, and higher groundwater usage—all of which disproportionately affect the poorer households. This finding highlights the need for a combination of decentralized sewerage options supported by local government and well-designed, installed, and maintained on-site sanitation systems if the risk of fecal exposure is to be sufficiently reduced for high-density, low-income communities.

In conclusion, on-site sanitation systems are likely to play a continuing integral role in most LMICs. To reach the ambition of the SDGs, significant changes are needed to on-site technology selection, implementation, and monitoring practices, including greater attention and tailored support mechanisms to ensure that inequalities in risk exposure are addressed.

## Figures and Tables

**Table 1:** Multivariate Poisson regression analysis on factors associated with septic tank emptying practices.

Tested Variables	*n* of Households	Adjusted Prevalence Ratio (95% CI)	*p*-Value
Administrative unit			<0.001
Municipality	26,786	1.07 (0.88–1.32)	0.483
District	109,439	Ref.	-
Population density			
Over 10,000 people/km^2^	4746	2.43 (1.80–3.28)	<0.001
1000–10,000 people/km^2^	33,127	2.14 (1.73–2.66)	<0.001
100–1000 people/km^2^	59,474	1.59 (1.30–1.95)	<0.001
Fewer than 100 people/km^2^	38,878	Ref.	-
Proportion of villages experiencing flood in a municipality or district			
Over 75%	1816	1.40 (1.06–1.85)	0.018
50%–75%	14,017	1.39 (1.09–1.77)	0.008
25%–50%	41,787	1.28 (1.09–1.50)	0.002
0%–25%	78,605	Ref.	-
Proportion of villages experiencing drought in a municipality or district			
Over 75%	0	-	-
50%–75%	846	0.14 (0.02–1.23)	0.077
25%–50%	11,348	0.46 (0.33–0.63)	<0.001
0%–25%	124,031	Ref.	-
Wealth quintile ^a^			
Richest	28,200	2.06 (1.90–2.23)	<0.001
Rich	30,821	1.51 (1.41–1.62)	<0.001
Middle	28,717	1.30 (1.21–1.39)	<0.001
Poor	25,844	1.23 (1.15–1.31)	<0.001
Poorest	22,643	Ref.	-
Sanitation facility			
Shared	9552	1.30 (1.22–1.38)	<0.001
Private	126,673	Ref.	-
Septic tank age			
Over 10 years old	61,738	5.20 (4.67–5.80)	<0.001
5–10 years old	34,157	2.31 (2.08–2.56)	<0.001
0–5 years old	40,330	Ref.	-
Size of household (scale)	136,225	1.08 (1.07–1.10)	<0.001

Wealth quintile ^a^: Wealth quintile calculations were based on reported expenditure per capita.

## Data Availability

Data are presented in the article and Supplementary Material.

## Supplementary Materials

The following are available online at https://www.mdpi.com/article/10.3390/ijerph18158204/s1, Figure S1. Septic tank and leach pit classification flow chart based on the national socio-economic survey questions for self-report septic tank households. Figure S2. Conceptual hierarchical model of factors for household septic tank emptying practice. Figure S3. Proportion of households reporting to practice septic tank emptying over proportion of households practicing open defecation. Each dot represents one municipality (grey circle, *n* = 99) or district (black circle, *n* = 415). Table S1. Municipality and district population density.

## References

[1] WHO, UNICEF (2017). Progress on Drinking Water, Sanitation and Hygiene: 2017 Update and SDG Baselines.

[2] Cumming O., Arnold B.F., Ban R., Clasen T.F., Mills J.E., Freeman M.C., Gordon B., Guiteras R., Howard G., Hunter P.R. (2019). The implications of three major new trials for the effect of water, sanitation and hygiene on childhood diarrhea and stunting: A consensus statement. BMC Med..

[3] Peal A., Evans B., Ahilan S., Ban R., Blackett I., Hawkins P., Schoebitz L., Scott R., Sleigh A., Strande L. (2020). Estimating Safely Managed Sanitation in Urban Areas; Lessons Learned from a Global Implementation of Excreta-Flow Diagrams. Front. Environ. Sci..

[4] Mitchell C., Abeysuriya K., Ross K., Information R. (2016). Making pathogen hazards visible: A new heuristic to improve sanitation investment efficacy. Waterlines.

[5] UNICEF, WHO (2019). PROGRESS on Household Drinking Water, Sanitation and Hygiene 2000–2017: Special Focus on Inequalities.

[6] Bain R., Johnston R., Mitis F., Chatterley C., Slaymaker T. (2018). Establishing Sustainable Development Goal Baselines for Household Drinking Water, Sanitation and Hygiene Services. Water.

[7] Berendes D.M., Sumner T.A., Brown J.M. (2017). Safely Managed Sanitation for All Means Fecal Sludge Management for At Least 1.8 Billion People in Low and Middle Income Countries. Environ. Sci. Technol..

[8] Strande L., Ronteltap M., Brdjanovic D. (2014). Faecal Sludge Management: Systems Approach for Implementation and Operation.

[9] WHO (2018). Guidelines on Sanitation and Health. https://apps.who.int/iris/bitstream/handle/10665/274939/9789241514705-eng.pdf.

[10] WHO (2019). Discussion Paper: Climate, Sanitation and Health. https://www.who.int/water_sanitation_health/sanitation-waste/sanitation/sanitation-and-climate-change20190813.pdf?ua=1.

[11] Mills F., Willetts J., Evans B., Carrard N., Kohlitz J. (2020). Costs, Climate and Contamination: Three Drivers for Citywide Sanitation Investment Decisions. Front. Environ. Sci..

[12] Frenoux C., Tsitsikalis A. (2014). Domestic private fecal sludge emptying services in Cambodia: Between market efficiency and regulation needs for sustainable management. J. Water Sanit. Hyg. Dev..

[13] Jenkins M.W., Cumming O., Cairncross S. (2015). Pit Latrine Emptying Behavior and Demand for Sanitation Services in Dar Es Salaam, Tanzania. Int. J. Environ. Res. Public Health.

[14] Odagiri M., Cronin A.A., Thomas A., Kurniawan M.A., Zainal M., Setiabudi W., Gnilo M.E., Badloe C., Virgiyanti T.D., Nurali I.A. (2020). Achieving the Sustainable Development Goals for water and sanitation in Indonesia—Results from a five-year (2013–2017) large-scale effectiveness evaluation. Int. J. Hyg. Environ. Health.

[15] National Bureau of Statistics Indonesia (BPS) (2018). Susenas (National Socio-Economic Survey).

[16] Ministry of Public Works and Housing (2018). Data Monitoring.

[17] Mills F. (2013). Assessment of Sludge Accumulation and Pit Filling Rates in Indonesia.

[18] Scott R., Ross I., Blackett I.C. (2016). Fecal Sludge Management: Diagnostics for Service Delivery in Urban Areas-Case Study in Balikpapan, Indonesia.

[19] Cronin A.A., Odagiri M., Arsyad B., Nuryetty M.T., Amannullah G., Santoso H., Darundiyah K., Nasution N. (2017). ’Aisyah Piloting water quality testing coupled with a national socioeconomic survey in Yogyakarta province, Indonesia, towards tracking of Sustainable Development Goal 6. Int. J. Hyg. Environ. Health.

[20] Afifah T., Nuryetty M.T., Cahyorini, Musadad D.A., Schlotheuber A., Bergen N., Johnston R. (2018). Subnational regional inequality in access to improved drinking water and sanitation in Indonesia: Results from the 2015 Indonesian National Socioeconomic Survey (SUSENAS). Glob. Health Action.

[21] National Standardization Agency of Indonesia (BSN) (2017). Indonesian National Standard—Standar Nasional Indonesia: Tata Cara Perencanaan Tangki Septik Dengan Pengolahan Lanjutan (Sumur Resapan, Bidang Resapan, Up Flow Filter, Kolam Sanita).

[22] Mills F., Blackett I.C., Tayler K. Assessing on-site systems and sludge accumulation rates to understand pit emptying in Indonesia. Sustainable water and sanitation services for all in a fast changing world. Proceedings of the 37th WEDC International Conference.

[23] Hathi P., Haque S., Pant L., Coffey D., Spears D. (2017). Place and Child Health: The Interaction of Population Density and Sanitation in Developing Countries. Demography.

[24] Fleming L., Anthonj C., Thakkar M.B., Tikoisuva W.M., Manga M., Howard G., Shields K.F., Kelly E., Overmars M., Bartram J. (2019). Urban and rural sanitation in the Solomon Islands: How resilient are these to extreme weather events?. Sci. Total Environ..

[25] Spears D. (2020). Exposure to open defecation can account for the Indian enigma of child height. J. Dev. Econ..

[26] Mills F., Willetts J., Petterson S., Mitchell C., Norman G. (2018). Faecal Pathogen Flows and Their Public Health Risks in Urban Environments: A Proposed Approach to Inform Sanitation Planning. Int. J. Environ. Res. Public Health.

[27] Wolf J., Johnston R., Hunter P.R., Gordon B., Medlicott K., Prüss-Ustün A. (2019). A Faecal Contamination Index for interpreting heterogeneous diarrhoea impacts of water, sanitation and hygiene interventions and overall, regional and country estimates of community sanitation coverage with a focus on low- and middle-income countries. Int. J. Hyg. Environ. Health.

[28] Zou G. (2004). A Modified Poisson Regression Approach to Prospective Studies with Binary Data. Am. J. Epidemiol..

[29] Carrard N., Foster T., Willetts J. (2019). Groundwater as a Source of Drinking Water in Southeast Asia and the Pacific: A Multi-Country Review of Current Reliance and Resource Concerns. Water.

[30] Graham J.P., Polizzotto M.L. (2013). Pit Latrines and Their Impacts on Groundwater Quality: A Systematic Review. Environ. Health Perspect..

[31] Lapworth D.J., Nkhuwa D.C.W., Okotto-Okotto J., Pedley S., Stuart M.E., Tijani M.N., Wright J. (2017). Urban groundwater quality in sub-Saharan Africa: Current status and implications for water security and public health. Hydrogeol. J..

[32] Borchardt M.A., Chyou P.-H., DeVries E.O., Belongia E.A. (2003). Septic system density and infectious diarrhea in a defined population of children. Environ. Health Perspect..

[33] Fong T.-T., Mansfield L.S., Wilson D.L., Schwab D.J., Molloy S.L., Rose J.B. (2007). Massive Microbiological Groundwater Contamination Associated with a Waterborne Outbreak in Lake Erie, South Bass Island, Ohio. Environ. Health Perspect..

[34] Daniels M.E., Smith W.A., Jenkins M.W. (2018). Estimating Cryptosporidium and Giardia disease burdens for children drinking untreated groundwater in a rural population in India. PLOS Neglected Trop. Dis..

[35] Ercumen A., Naser A.M., Arnold B.F., Unicomb L., Colford J.M., Luby S.P. (2017). Can sanitary inspection surveys predict risk of microbiological contamination of groundwater sources?. Evidence from shallow tubewells in rural Bangladesh. Am. J. Trop. Med. Hyg..

[36] Ravenscroft P., Mahmud Z.H., Islam M.S., Hossain A., Zahid A., Saha G., Ali A.Z., Islam K., Cairncross S., Clemens J. (2017). The public health significance of latrines discharging to groundwater used for drinking. Water Res..

[37] Escamilla V., Knappett P.S.K., Yunus M., Streatfield P.K., Emch M. (2013). Influence of Latrine Proximity and Type on Tubewell Water Quality and Diarrheal Disease in Bangladesh. Ann. Assoc. Am. Geogr..

[38] Nielson, UNICEF (2020). Market Assessment for Safely Managed Sanitation in Indonesia.

[39] Verhagen J., Scott P. (2019). Safely Managed Sanitation in High Density Rural Areas: Turning Fecal Sludge into a Resource through Innovative Waste Management.

[40] WaterSHED (2018). Behavioral Drivers of Fecal Sludge Management in Rural Cambodia: A Qualitative Study.

[41] Mehta M., Mehta D., Yadav U. (2019). Citywide Inclusive Sanitation Through Scheduled Desludging Services: Emerging Experience from India. Front. Environ. Sci..

[42] Greene N., Hennessy S., Rogers T.W., Tsai J., de los Reyes F.L. (2021). The role of emptying services in provision of safely managed sanitation: A classification and quantification of the needs of LMICs. J. Environ. Manag..

[43] Amin N., Liu P., Foster T., Rahman M., Miah R., Ahmed G.B., Kabir M., Raj S., Moe C.L., Willetts J. (2020). Pathogen flows from on-site sanitation systems in low-income urban neighborhoods, Dhaka: A quantitative environmental assessment. Int. J. Hyg. Environ. Health.

[44] Berendes D.M., Leon J.S., Kirby A.E., Clennon J.A., Raj S.J., Yakubu H., Robb K.A., Kartikeyan A., Hemavathy P., Gunasekaran A. (2019). Associations between open drain flooding and pediatric enteric infections in the MAL-ED cohort in a low-income, urban neighborhood in Vellore, India. BMC Public Health.

[45] Berendes D.M., Kirby A.E., Clennon J.A., Agbemabiese C., Ampofo J.A., Armah G.E., Baker K., Liu P., Reese H.E., Robb K.A. (2018). Urban sanitation coverage and environmental fecal contamination: Links between the household and public environments of Accra, Ghana. PLoS ONE.

[46] Daniels M., Smith W.A., Schmidt W.-P., Clasen T.F., Jenkins M.W. (2016). Modeling Cryptosporidium and Giardia in Ground and Surface Water Sources in Rural India: Associations with Latrines, Livestock, Damaged Wells, and Rainfall Patterns. Environ. Sci. Technol..

[47] Willetts J., Mills F., Al’Afghani M. (2020). Sustaining Community-Scale Sanitation Services: Co-management by Local Government and Low-Income Communities in Indonesia. Front. Environ. Sci..

[48] Hashemi S. (2020). Sanitation Sustainability Index: A Pilot Approach to Develop a Community-Based Indicator for Evaluating Sustainability of Sanitation Systems. Sustainability.

[49] UNICEF What Do Safely Managed Sanitation Services Mean for UNICEF Programmes?. 2020..

[50] Harper J., Bielefeldt A., Javernick-Will A., Veasna T., Nicoletti C. (2020). Context and intentions: Practical associations for fecal sludge management in rural low-income Cambodia. J. Water Sanit. Hyg. Dev..

[51] Myers J., Bongartz P., Vernon N., Fox J. (2016). Chapter 8: The long-term safe management of rural shit. Sustainable Sanitation for All: Experiences, Challenges, and Innovations.

[52] Jenkins M.W., Cumming O., Scott B., Cairncross S. (2014). Beyond ‘improved’ towards ‘safe and sustainable’ urban sanitation: Assessing the design, management and functionality of sanitation in poor communities of Dar es Salaam, Tanzania. J. Water Sanit. Hyg. Dev..

[53] Odagiri M., Muhammad Z., Cronin A.A., Gnilo M.E., Mardikanto A.K., Umam K., Asamou Y.T. (2017). Enabling Factors for Sustaining Open Defecation-Free Communities in Rural Indonesia: A Cross-Sectional Study. Int. J. Environ. Res. Public Health.

[54] Myers J., Maule L., Gnilo M., Chambers R., Cavill S. (2017). Supporting the Least Able Throughout and Beyond CLTS.

[55] Kohlitz J., Carrard N., Willetts J. (2019). Support Mechanisms to Strengthen Equality and Non-Discrimination (EQND) in Rural Sanitation (Part 2 of 2).

[56] Schrecongost A., Pedi D., Rosenboom J.W., Shrestha R., Ban R. (2020). Citywide Inclusive Sanitation: A Public Service Approach for Reaching the Urban Sanitation SDGs. Front. Environ. Sci..

[57] Carrard N., Kohlitz J., Soeters S., Halcrow G., Murta J., Willetts J. (2020). Reaching all in rural sanitation: Experiences from inclusive programming in five countries. Dev. Pract..

[58] Khan S.M., Bain R.E.S., Lunze K., Unalan T., Beshanski-Pedersen B., Slaymaker T., Johnston R., Hancioglu A. (2017). Optimizing household survey methods to monitor the Sustainable Development Goals targets 6.1 and 6.2 on drinking water, sanitation and hygiene: A mixed-methods field-test in Belize. PLoS ONE.

