# Supplementary material for: Safely Managed On-Site Sanitation: A National Assessment of Sanitation Services and Potential Fecal Exposure in Indonesia
Source: Int J Environ Res Public Health. 2021 Aug 3;18(15):8204. doi: 10.3390/ijerph18158204 (PMC8346009; doi:10.3390/ijerph18158204)
Supplement: Supplementary file 1 [file ijerph-18-08204-s001.zip › ijerph-1278647-SI.pdf]

Supplemental Materials:

Is it safely managed? A national assessment of on-site sanitation services and potential fecal exposure in Indonesia

Mitsunori Odagiri[1], Ann Thomas[1], Maraita Listyasari[1], Freya Mills[2], Robert Bain[3], Muhammad Zainal[1], Tom Slaymaker[3], Aldy Mardikanto[4], Anita Gultom[5], Asri Indiyani[6], Hasnani Rangkuti[7], Juliet Willetts[2]

[1] UNICEF Indonesia, Indonesia
[2] Institute for Sustainable Futures, University of Technology Sydney, Australia
[3] UNICEF New York, USA
[4] National Development Planning Agency (Bappenas), Government of Indonesia, Indonesia
[5] Ministry of Health, Government of Indonesia, Indonesia
[6] Ministry of Public Work and Housing, Government of Indonesia, Indonesia
[7] National Bureau of Statistics (BPS), Government of Indonesia, Indonesia

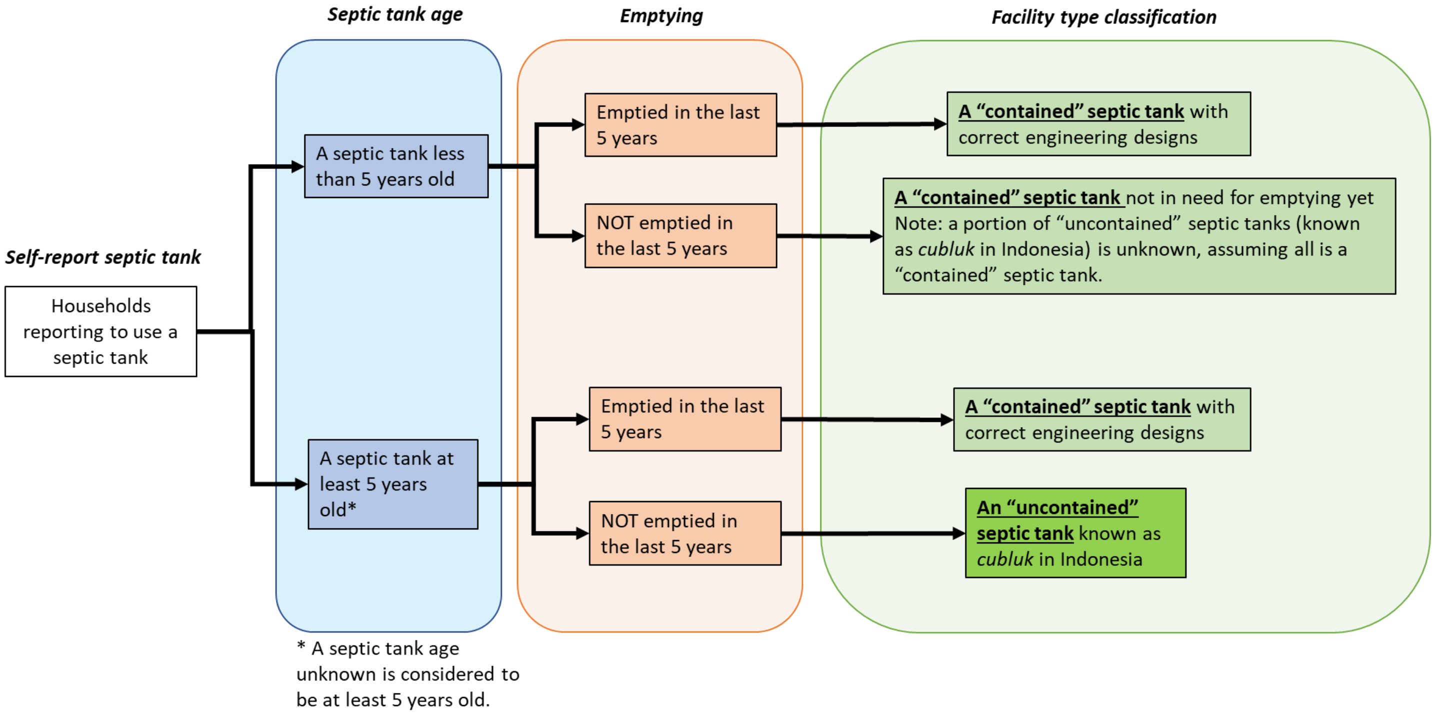

Figure S1. Septic tank and leach pit classification flow chart based on the national socio-economic survey questions for self-report septic tank households.

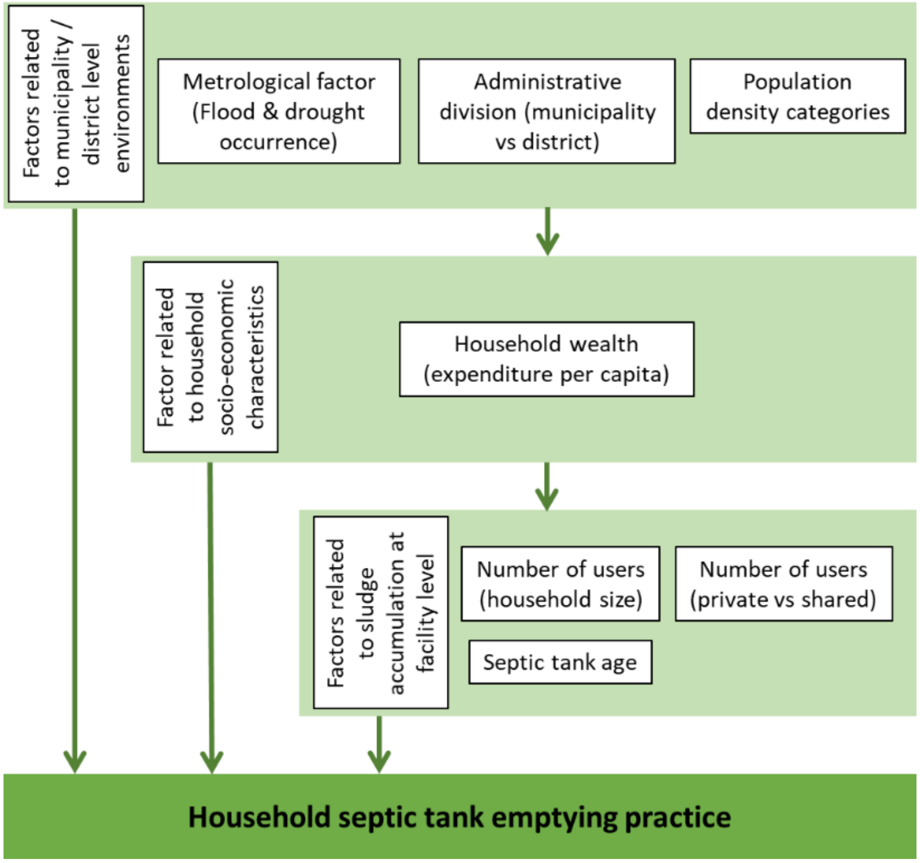

Figure S2. Conceptual hierarchical model of factors for household septic tank emptying practice

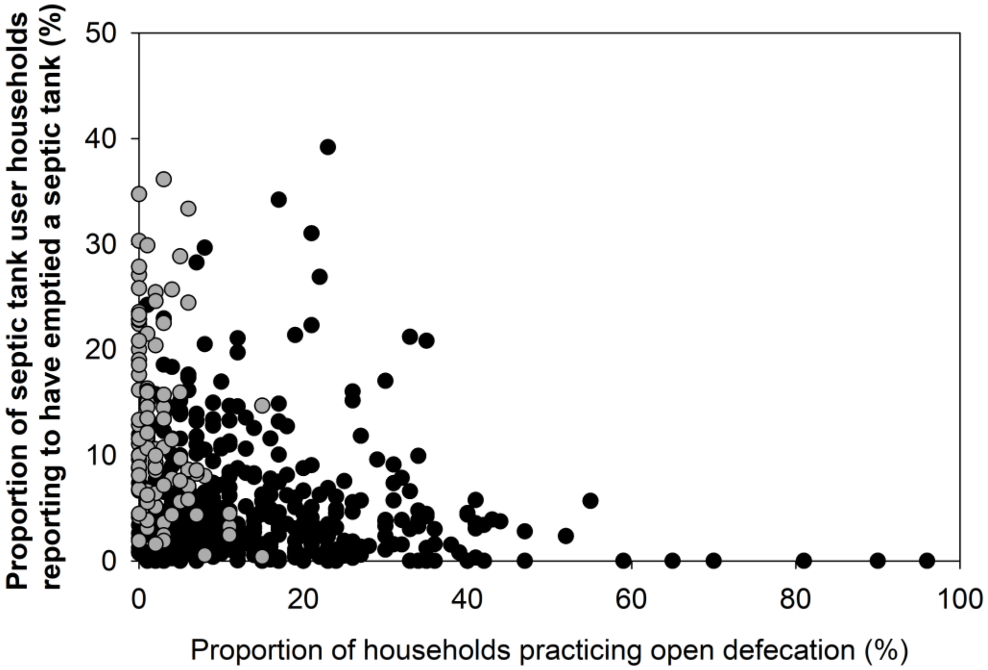

Figure S3. Proportion of households reporting to practice septic tank emptying over proportion of households practicing open defecation. Each dot represents one municipality (grey circle, n = 99) or district (black circle, n = 415).

Table S1. Municipality and district population density

Name of municipalities and districts	Population density (people / km2)
Aceh Barat	71.8
Aceh Barat Daya	100.9
Aceh Besar	143.2
Aceh Jaya	24.4
Aceh Selatan	62.0
Aceh Singkil	56.8
Aceh Tamiang	150.8
Aceh Tengah	49.2
Aceh Tenggara	51.2
Aceh Timur	69.4
Aceh Utara	191.4
Agam	272.3
Alor	71.8
Asahan	197.1
Asmat	3.0
Badung	1601.0
Balangan	70.0
Bandung	2135.4
Bandung Barat	1301.8
Banggai	39.0
Banggai Kepulauan	47.6
Banggai Laut	103.4
Bangka	114.3
Bangka Barat	75.6
Bangka Selatan	58.2
Bangka Tengah	91.0
Bangkalan	985.3
Bangli	463.2
Banjar	126.0
Banjarnegara	901.8
Bantaeng	474.0
Bantul	2004.2
Banyu Asin	72.2
Banyumas	1267.9
Banyuwangi	279.1
Barito Kuala	104.7
Barito Selatan	15.5
Barito Timur	33.1
Barito Utara	15.7
Barru	148.4
Batang	974.6
Batang Hari	47.0
Batu Bara	451.6

Name of municipalities and districts	Population density (people / km2)
Kota Tasikmalaya	3866.4
Kota Tebing Tinggi	5303.3
Kota Tegal	6298
Kota Ternate	2093.6
Kota Tidore Kepulauan	61.6
Kota Tomohon	936.2
Kota Tual	297.1
Kota Yogyakarta	13290.4
Kotawaringin Barat	29.1
Kotawaringin Timur	27.8
Kuantan Singingi	62.2
Kubu Raya	83.3
Kudus	2049.4
Kulon Progo	733.8
Kuningan	973.2
Kupang	74.3
Kutai Barat	7.3
Kutai Kartanegara	33.3
Kutai Timur	10.5
Labuhan Batu	229.2
Labuhan Batu Selatan	94.3
Labuhan Batu Utara	101.9
Lahat	77.1
Lamandau	12.9
Lamongan	667.3
Lampung Barat	141.3
Lampung Selatan	1444.0
Lampung Tengah	336.9
Lampung Timur	196.1
Lampung Utara	226.3
Landak	42.3
Langkat	166.4
Lanny Jaya	79.6
Lebak	380.2
Lebong	60.7
Lembata	113.0
Lima Puluh Kota	107.2
Lingga	39.6
Lombok Barat	775.2
Lombok Tengah	865.3
Lombok Timur	975.5
Lombok Utara	283.9
Lumajang	582.1

Bekasi	3072.9
Belitung	82.8
Belitung Timur	51.7
Belu	171.3
Bener Meriah	101.9
Bengkalis	353.0
Bengkayang	50.3
Bengkulu Selatan	133.6
Bengkulu Tengah	93.7
Bengkulu Utara	71.7
Berau	10.9
Biak Numfor	58.6
Bima	143.5
Bintan	120.9
Bireuen	248.1
Blitar	868.5
Blora	479.3
Boalemo	109.7
Bogor	2200.8
Bojonegoro	568.4
Bolaang Mongondow	86.3
Bolaang Mongondow Selatan	40.9
Bolaang Mongondow Timur	79.6
Bolaang Mongondow Utara	47.8
Bombana	61.5
Bondowoso	508.3
Bone	166.4
Bone Bolango	81.3
Boven Digoel	2.6
Boyolali	976.6
Brebes	951.0
Buleleng	484.0
Bulukumba	327.4
Bulungan	10.2
Bungo	80.4
Buol	40.1
Buru	29.1
Buru Selatan	16.8
Buton	84.6
Buton Selatan	158.4
Buton Tengah	97.2
Buton Utara	34.4
Ciamis	844.8
Cianjur	589.3
Cilacap	813.0
Cirebon	2227.4

Luwu	108.3
Luwu Timur	43.2
Luwu Utara	41.7
Madiun	658.0
Magelang	1170.1
Magetan	913.1
Mahakam Ulu	1.7
Majalengka	1000.7
Majene	183.5
Malaka	165.3
Malang	738.2
Malinau	2.1
Maluku Barat Daya	16.0
Maluku Tengah	46.9
Maluku Tenggara	96.7
Maluku Tenggara Barat	25.3
Mamasa	53.9
Mamberamo Raya	1.0
Mamberamo Tengah	37.8
Mamuju	58.7
Mamuju Tengah	44.5
Mamuju Utara	57.3
Mandailing Natal	72.9
Manggarai	161.4
Manggarai Barat	114.6
Manggarai Timur	108.7
Manokwari	55.0
Manokwari Selatan	8.6
Mappi	4.3
Maros	218.1
Maybrat	7.5
Melawi	19.6
Mempawah (Kab. Pontianak)	94.4
Merangin	50.6
Merauke	5.2
Mesuji	91.7
Mimika	10.2
Minahasa	306.0
Minahasa Selatan	149.4
Minahasa Tenggara	150.4
Minahasa Utara	221.7
Mojokerto	1557
Morowali	39.9
Morowali Utara	12.8
Muara Enim	86.2
Muaro Jambi	83.2

Dairi	147.5
Deiyai	136.2
Deli Serdang	979.5
Demak	1291.8
Dharmas Raya	83.6
Dogiyai	23.1
Dompu	105.5
Donggala	71.1
Empat Lawang	110.9
Ende	133.9
Enrekang	115.6
Fakfak	7.1
Flores Timur	141.1
Garut	853.1
Gayo Lues	16.5
Gianyar	1391.8
Gorontalo	216.2
Gorontalo Utara	68.7
Gowa	410.3
Gresik	1102.1
Grobogan	684.2
Gunung Kidul	518.9
Gunung Mas	11.1
Halmahera Barat	69.4
Halmahera Tengah	21.0
Halmahera Timur	14.5
Halmahera Utara	49.7
Halmaltera Selatan	28.8
Hulu Sungai Selatan	131.7
Hulu Sungai Tengah	185.1
Hulu Sungai Utara	266.1
Humbang Hasundutan	81.4
Indragiri Hilir	106.2
Indragiri Hulu	57.2
Indramayu	847.2
Intan Jaya	12.6
Jayapura	11.8
Jayawijaya	31.0
Jember	792.5
Jembrana	330.4
Jeneponto	514.9
Jepara	1187.5
Jombang	1133.4
Kaimana	3.7
Kampar	79.3
Kapuas	23.9

Mukomuko	48.0
Muna	118.3
Muna Barat	76.7
Murung Raya	5.1
Musi Banyuasin	45.4
Musi Rawas	63.6
Musi Rawas Utara	32.0
Nabire	13.5
Nagan Raya	49.7
Nagekeo	102.9
Natuna	38.7
Nduga	45.5
Ngada	99.2
Nganjuk	861.4
Ngawi	640.5
Nias	75.3
Nias Barat	183.0
Nias Selatan	175.3
Nias Utara	114.7
Nunukan	15.2
Ogan Ilir	161.3
Ogan Komering Ilir	45.2
Ogan Komering Ulu	76.7
Ogan Komering Ulu Selatan	65.7
Ogan Komering Ulu Timur	200.8
Pacitan	399.5
Padang Lawas	72.2
Padang Lawas Utara	69.6
Padang Pariaman	311.9
Pakpak Bharat	40.2
Pamekasan	1110.8
Pandeglang	441.2
Pangandaran	395.3
Pangkajene Kepulauan	296.4
Paniai	27.2
Parigi Moutong	96.4
Pasaman	71.2
Pasaman Barat	114.1
Paser	37.0
Pasuruan	1104.1
Pati	845.8
Pegunungan Arfak	11.2
Pegunungan Bintang	4.8
Pekalongan	1072.5
Pelalawan	37.9
Pemalang	1165.3

Kapuas Hulu	8.8
Karang Asem	496.3
Karanganyar	1143.2
Karawang	1424.7
Karimun	255.1
Karo	195.5
Katingan	9.7
Kaur	51.2
Kayong Utara	24.7
Kebumen	988.6
Kediri	1135.8
Keerom	6.8
Kendal	868.5
Kepahiang	206.3
Kepulauan Anambas	71.7
Kepulauan Aru	11.8
Kepulauan Mentawai	15.3
Kepulauan Meranti	50.0
Kepulauan Sangihe	284.5
Kepulauan Seribu	2386.5
Kepulauan Sula	31.1
Kepulauan Talaud	74.6
Kerinci	71.1
Ketapang	16.4
Klaten	1785.1
Klungkung	568.5
Kolaka	61.6
Kolaka Timur	53.1
Kolaka Utara	44.5
Konawe	57.4
Konawe Kepulauan	39.4
Konawe Selatan	54.5
Konawe Utara	12.5
Kota Ambon	1602.8
Kota Balikpapan	1243.2
Kota Banda Aceh	4405.5
Kota Bandar Lampung	3552.4
Kota Bandung	14957.3
Kota Banjar	1613.4
Kota Banjar Baru	708.1
Kota Banjarmasin	9841.8
Kota Baru	36.1
Kota Batam	1433.0
Kota Batu	1517.4
Kota Bau-Bau	777.4
Kota Bekasi	14539.1

Penajam Paser Utara	48.3
Penukal Abab Lematang Ilir	103.1
Pesawaran	198.1
Pesisir Barat	53.3
Pesisir Selatan	80.7
Pidie	144.1
Pidie Jaya	150.2
Pinrang	192.2
Pohuwato	38.0
Poliwali Mandar	249.2
Ponorogo	667.4
Poso	36.0
Pringsewu	640.3
Probolinggo	688.9
Pulang Pisau	14.1
Pulau Morotai	27.2
Pulau Taliabu	36.1
Puncak	14.1
Puncak Jaya	25.9
Purbalingga	1378.5
Purwakarta	1166.1
Purworejo	658.1
Raja Ampat	6.0
Rejang Lebong	159.1
Rembang	719.4
Rokan Hilir	80.4
Rokan Hulu	91.2
Rote Ndao	134.5
Sabu Raijua	211.4
Sambas	79.8
Samosir	61.0
Sampang	793.8
Sanggau	36.6
Sarmi	2.3
Sarolangun	48.8
Sekadau	37.0
Selayar	99.9
Seluma	80.7
Semarang	1109
Seram Bagian Barat	34.1
Seram Bagian Timur	17.8
Serang	869.8
Serdang Bedagai	324.4
Seruyan	12.6
Siak	59.2
Siau Tagulandang Biaro	240.7

Kota Bengkulu	2538.8
Kota Bima	778.5
Kota Binjai	4673.0
Kota Bitung	723.0
Kota Blitar	4356.0
Kota Bogor	9384.6
Kota Bontang	437.0
Kota Bukittinggi	5181.2
Kota Cilegon	2491.2
Kota Cimahi	15643.1
Kota Cirebon	8546.9
Kota Denpasar	7412.1
Kota Depok	12016.7
Kota Dumai	190.2
Kota Gorontalo	2756.6
Kota Gunungsitoli	507.3
Kota Jakarta Barat	20812.7
Kota Jakarta Pusat	17718.8
Kota Jakarta Selatan	14675.3
Kota Jakarta Timur	16080.2
Kota Jakarta Utara	12950.3
Kota Jambi	5840.6
Kota Jayapura	320.7
Kota Kediri	4533.3
Kota Kendari	1305.6
Kota Kotamobagu	1886.4
Kota Kupang	16614.7
Kota Langsa	673.8
Kota Lhokseumawe	1144.4
Kota Lubuklinggau	578.4
Kota Madiun	5218.4
Kota Magelang	7603.4
Kota Makassar	7661.7
Kota Malang	5993.1
Kota Manado	2757.3
Kota Mataram	7939.9
Kota Medan	8603.4
Kota Metro	2709.4
Kota Mojokerto	7833.3
Kota Padang	1370.8
Kota Padang Panjang	2334.5
Kota Padangsidimpuan	1934.7
Kota Pagar Alam	219.7
Kota Palangka Raya	121.6
Kota Palembang	4503.8
Kota Palopo	729.7

Sidenreng Rappang	160.3
Sidoarjo	3545.9
Sigi	46.1
Sijunjung	75.8
Sikka	185.0
Simalungun	198.7
Simeulue	45.4
Sinjai	305.6
Sintang	19.4
Situbondo	409.0
Sleman	2121.8
Solok	99.9
Solok Selatan	51.1
Soppeng	145.8
Sorong	13.6
Sorong Selatan	7.1
Sragen	945.8
Subang	842.6
Sukabumi	594.9
Sukamara	16.8
Sukoharjo	1823.5
Sumba Barat	59.4
Sumba Barat Daya	232.8
Sumba Tengah	39.0
Sumba Timur	36.9
Sumbawa	68.9
Sumbawa Barat	80.4
Sumedang	759.0
Sumenep	544.9
Supiori	30.5
Tabalong	67.5
Tabanan	439.6
Takalar	527.1
Tambraw	1.2
Tana Tidung	6.0
Tana Toraja	117.6
Tanah Bumbu	71.9
Tanah Datar	260.6
Tanah Laut	94.7
Tangerang	3756.2
Tanggamus	198.1
TanjungJabung Barat	71.8
TanjungJabung Timur	40.4
Tapanuli Selatan	46.8
Tapanuli Tengah	172.2
Tapanuli Utara	79.6

Kota Palu	990.7
Kota Pangkal Pinang	2379.5
Kota Pare-Pare	1461.6
Kota Pariaman	1338.3
Kota Pasuruan	5679.3
Kota Payakumbuh	1590.9
Kota Pekalongan	6786.7
Kota Pekanbaru	1808.3
Kota Pematang Siantar	4587.1
Kota Pontianak	5998.7
Kota Prabumulih	741.6
Kota Probolinggo	4185.8
Kota Sabang	227.9
Kota Salatiga	3383.6
Kota Samarinda	1114.6
Kota Sawah Lunto	269.6
Kota Semarang	4853.4
Kota Serang	2581.8
Kota Sibolga	2121.2
Kota Singkawang	442.3
Kota Solok	996.1
Kota Sorong	387.1
Kota Subulussalam	58.5
Kota Sukabumi	6812
Kota Sungai Penuh	232.2
Kota Surabaya	8262.1
Kota Surakarta	11292.9
Kota Tangerang	14486.5
Kota Tangerang Selatan	11875.2
Kota Tanjung Balai	1625.0
Kota Tanjung Pinang	1463.6
Kota Tarakan	1080.1

Tapin	70.9
Tasikmalaya	687.6
Tebo	54.9
Tegal	1644.4
Teluk Bintuni	3.1
Teluk Wondama	8.2
Temanggung	921.6
Timor Tengah Selatan	118.6
Timor Tengah Utara	95.2
Toba Samosir	78.9
Tojo Una-Una	26.9
Toli-Toli	57.8
Tolikara	24.9
Toraja Utara	190.2
Trenggalek	606.9
Tuban	639.4
Tulangbawang	130.1
Tulangbawang Barat	227.5
Tulungagung	984.5
Wajo	158.9
Wakatobi	171.4
Waropen	2.9
Way Kanan	114.8
Wonogiri	534.9
Wonosobo	805.5
Yahukimo	11.1
Yalimo	50.0
Yapen Waropen	49.4
